# Supplementary material for: Occurrence of Chlamydia spp. in Conjunctival Samples of Stray Cats in Timișoara Municipality, Western Romania
Source: Microorganisms. 2022 Nov 4;10(11):2187. doi: 10.3390/microorganisms10112187 (PMC9693150; doi:10.3390/microorganisms10112187)
Supplement: Supplementary file 1 [file microorganisms-10-02187-s001.zip › Supplementary Table S1.pdf]

Results of descriptive statistic:

Sex

		Frequency	Percent	Valid Percent	Cumulative Percent
Valid	Male	55	57,9	57,9	57,9
	Female	40	42,1	42,1	100,0
	Total	95	100,0	100,0	

Age

		Frequency	Percent	Valid Percent	Cumulative Percent
Valid	< 2 months	26	27,4	27,4	27,4
	> 2 months and < 6 months	31	32,6	32,6	60,0
	> 6 months and < 2 years	30	31,6	31,6	91,6
	> 2 years	8	8,4	8,4	100,0
	Total	95	100,0	100,0	

Type

		Frequency	Percent	Valid Percent	Cumulative Percent
Valid	Symptomatic cats	27	28,4	28,4	28,4
	Asymptomatic cats	68	71,6	71,6	100,0
	Total	95	100,0	100,0	

Type

		Frequency	Percent	Valid Percent	Cumulative Percent
Valid	Symptomatic cats	27	28,4	28,4	28,4
	Asymptomatic cats	68	71,6	71,6	100,0
	Total	95	100,0	100,0	

Positive

		Frequency	Percent	Valid Percent	Cumulative Percent
Valid	Synptomatic cats Positive PCR for Chlamydia	17	17,9	17,9	17,9
	Asynptomatic cats Positive PCR for Chlamydia	46	48,4	48,4	66,3
	Negative	32	33,7	33,7	100,0
	Total	95	100,0	100,0	

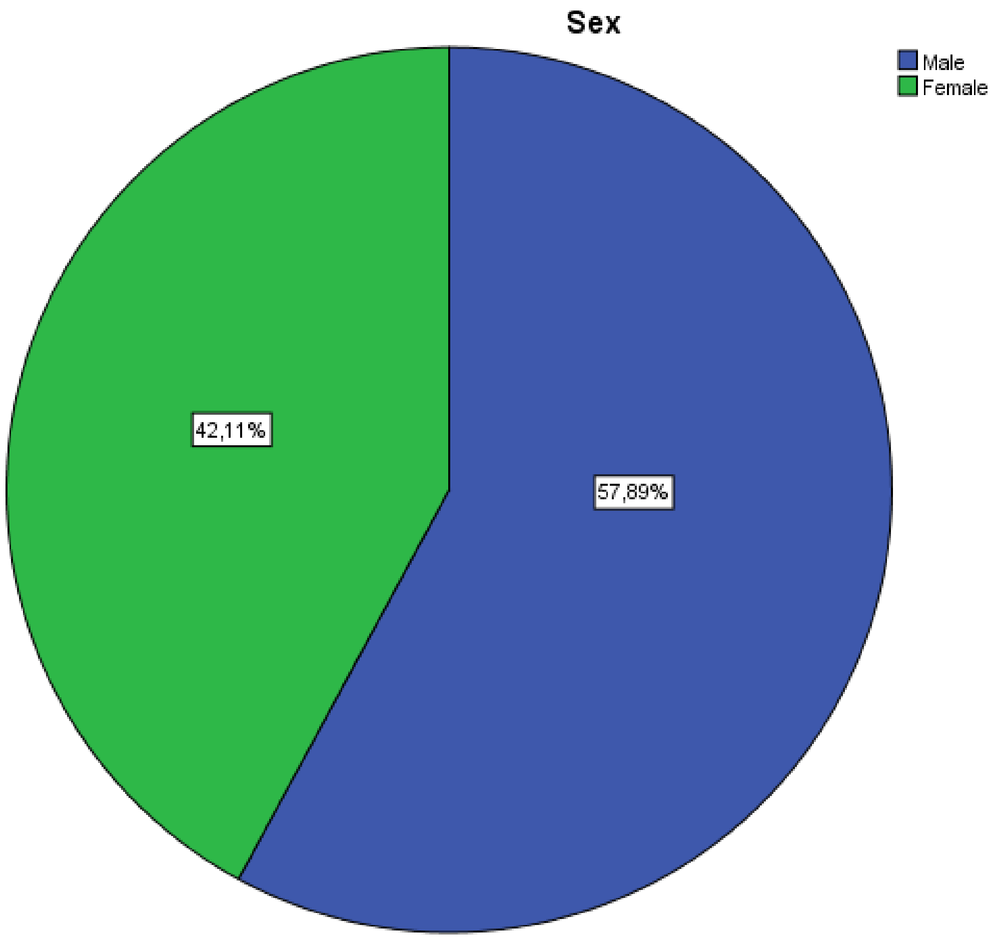

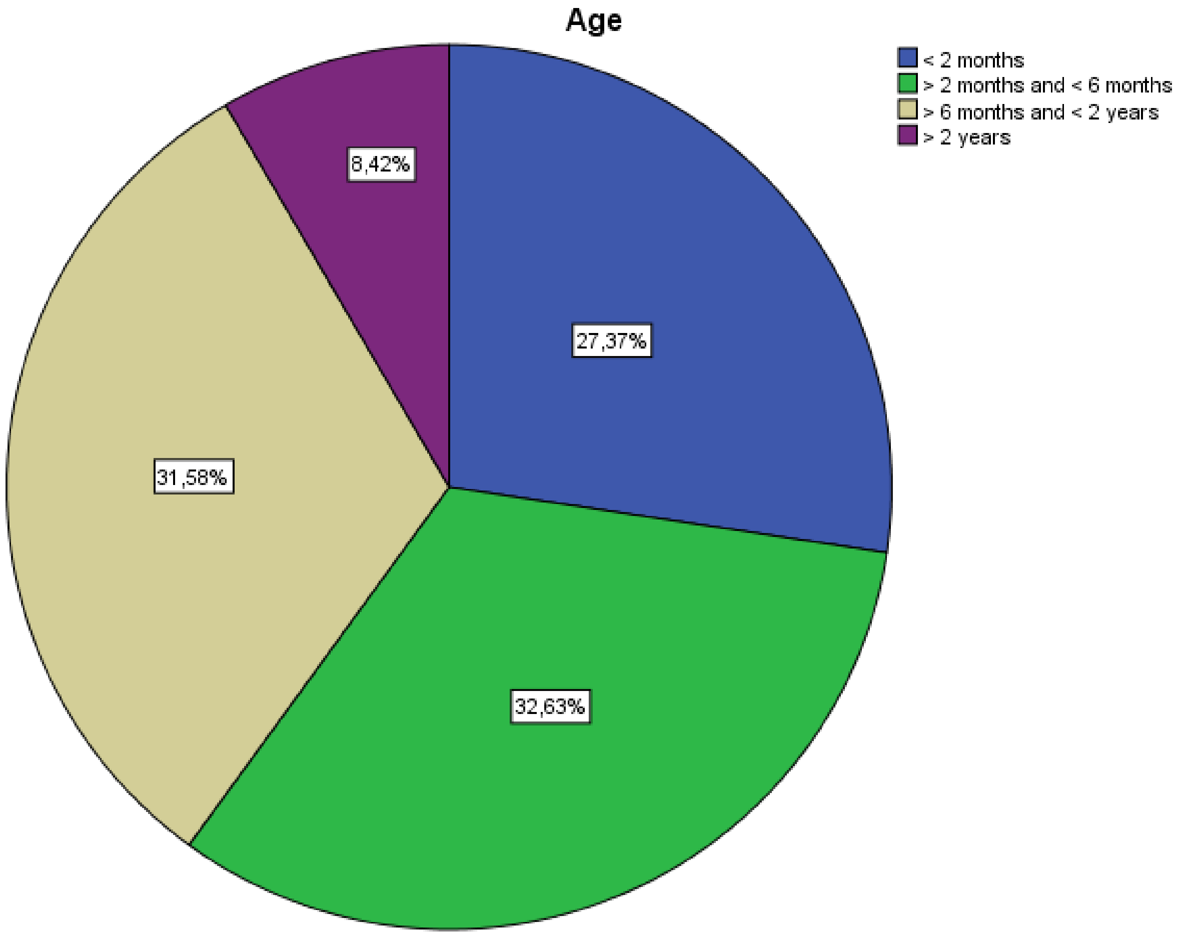
Age
< 2 months
> 2 months and < 6 months
> 6 months and < 2 years
> 2 years
27,37%
32,63%
31,58%
8,42%

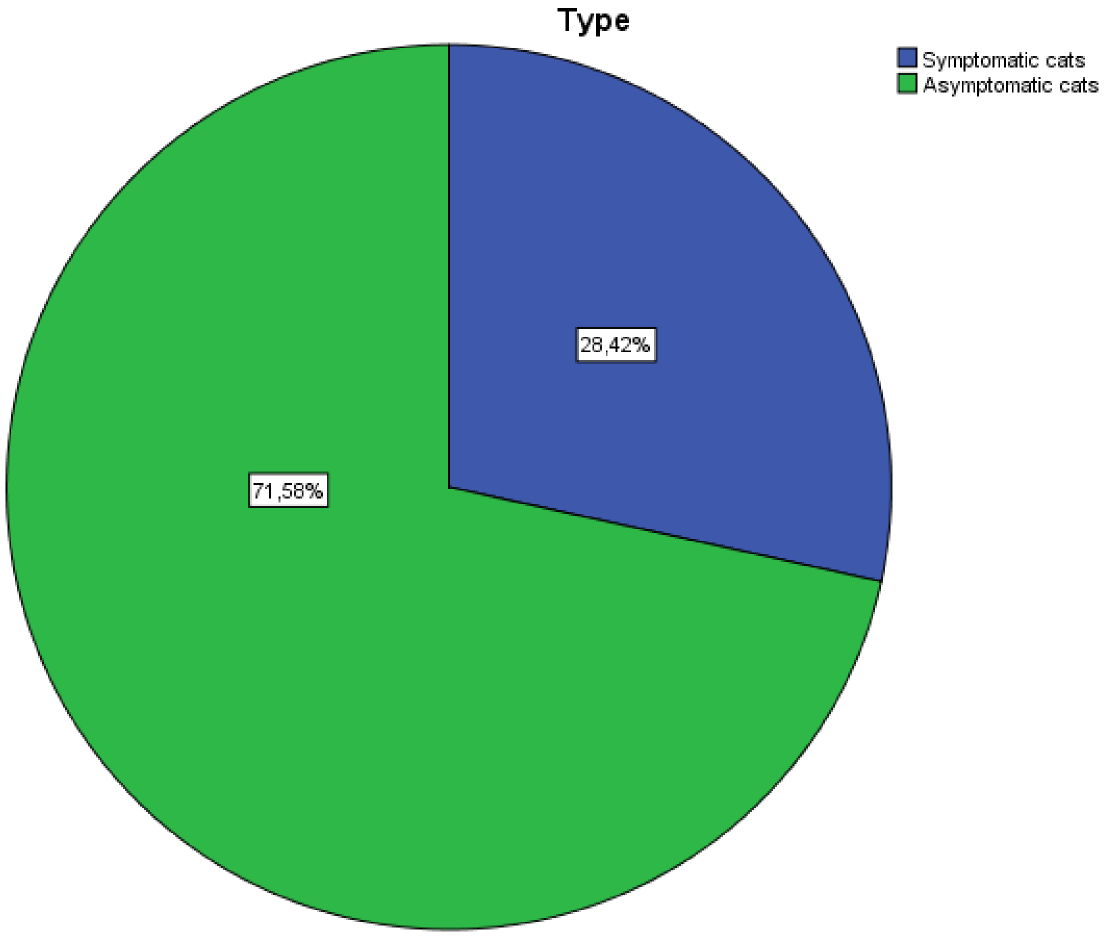
Type
Symptomatic cats
Asymptomatic cats
28,42%
71,58%

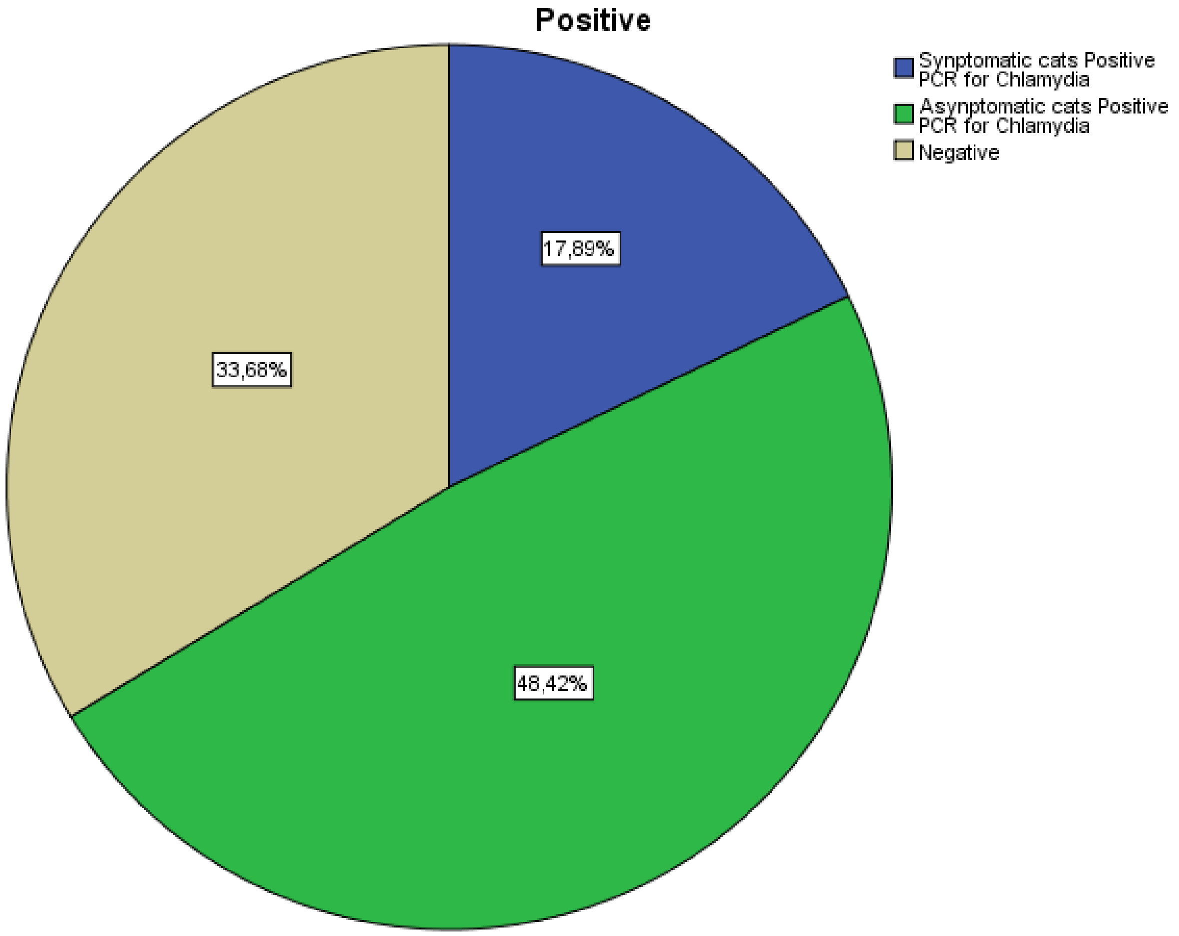

Positive
Synptomatic cats Positive PCR for Chlamydia
Asynptomatic cats Positive PCR for Chlamydia
Negative
17,89%
33,68%
48,42%
